# Cultural adaptation and validation of the Arabic version of the short 12-item stroke-specific quality of life scale

**DOI:** 10.3389/fneur.2023.1232602

**Published:** 2023-10-09

**Authors:** Fouad Sakr, Mariam Dabbous, Marwan Akel, Pascale Salameh, Hassan Hosseini

**Affiliations:** ^1^École Doctorale Sciences de la Vie et de la Santé, Université Paris-Est Créteil, Créteil, France; ^2^UMR U955 INSERM, Institut Mondor de Recherche Biomédicale, Université Paris-Est Créteil, Créteil, France; ^3^School of Pharmacy, Lebanese International University, Beirut, Lebanon; ^4^INSPECT-LB: Institut National de Santé Publique, Épidémiologie Clinique et Toxicologie-Liban, Beirut, Lebanon; ^5^International Pharmaceutical Federation (FIP), The Hague, Netherlands; ^6^Faculty of Public Health, Lebanese University, Beirut, Lebanon; ^7^University of Nicosia Medical School, Nicosia, Cyprus; ^8^School of Medicine, Lebanese American University, Byblos, Lebanon; ^9^Stroke Unit, Service de Neurologie, CHU Henri Mondor, Créteil, France

**Keywords:** stroke, stroke-specific, validated scale, quality of life, SS-QOL, short scale

## Abstract

**Background:**

Given the excessive length and inconsistent validity of the existing long stroke-specific quality of life (SS-QOL) scales, there is a need to validate a shorter measuring tool. The aim of this study was to validate the Arabic version of the short 12-item SS-QOL (SS-QOL-12-AR) and examine its validity measures and psychometric properties. Additionally, the study aimed to assess the QOL after stroke and identify the sociodemographic and clinical factors that influence it in Lebanon.

**Methods:**

A cross-sectional study was conducted. The SS-QOL-12-AR structure was validated, and its reliability and internal consistency were assessed. The scale’s specificity and sensitivity were evaluated and then compared with those of other SS-QOL scales. The correlation between each item and the overall scale were examined, and its convergent validity was evaluated.

**Results:**

A total of 172 stroke survivors were included. The SS-QOL-12-AR structure was validated with a solution of two factors, with a Kaiser-Meyer-Olkin measure of sampling adequacy of 0.850 and a significant Bartlett’s test of sphericity (*p* < 0.001). The Cronbach’s alpha of the scale was 0.917. According to ROC curve analysis, the optimal cut-off point for distinguishing between lower and better QOL was 32.50. At this cut-off, the sensitivity and specificity were 70.0% and 71.2%, respectively. The area under the curve was 0.779 (95% CI 0.704–0.855, *p* < 0.001). The SS-QOL-12-AR demonstrated a strong and highly significant correlation with existing versions of the SS-QOL, confirming its convergent validity. 61.6% of stroke survivors had a lower QOL, which was significantly associated with poor stroke prognosis, increased physical dependence, current smoking, and alcohol abstinence.

**Conclusion:**

The SS-QOL-12-AR exhibits strong validity and reliability, demonstrating excellent psychometric properties. The scale holds potential for application in clinical practice and research settings, enabling the measurement of stroke-related consequences and evaluation of management outcomes.

## 1. Introduction

Stroke is a significant contributor to global disability, morbidity, and mortality (1). It exerts a profound influence on the well-being of survivors, who often grapple with enduring consequences (2). Long-term sequelae following a stroke encompass an array of challenges, spanning functional limitations, mental health issues, social obstacles, and emotional distress (3). Merely evaluating disability and impairment is inadequate in comprehending the full extent of stroke’s influence. Increasingly, there is a realization of the crucial significance of functional outcome indicators in post-stroke care and rehabilitation. In this regard, the evaluation of health-related quality of life (HRQOL) is increasingly employed as a progressive means to gauge the situation of stroke survivors (4). Consequently, the assessment of HRQOL has gained considerable prominence and widespread acceptance as a fundamental tool in appraising chronic medical conditions such as stroke. By scrutinizing aspects that patients hold value, this evaluation takes into account how their health status influences their ability to lead a satisfactory life (5).

Patients’ perception of quality of life (QOL) varies based on their unique objectives, disquiets, desires, cultural background, and value systems, highlighting the subjective nature of this construct (6). In measuring stroke outcomes, two primary objectives have emerged, delineating the repercussions of stroke and evaluating stroke care (7). To accomplish this, generic measures of HRQOL have predominantly been employed. These measures were originally designed to assess the impact of common health conditions on daily functioning within the general population. Notable examples include the Short Form-6D (derived from the Short Form-36), the EuroQol-5D (EQ-5D), and the Health Utilities Index (8–10). Although these generic measures have found utility in stroke research, it is crucial to acknowledge their fundamental limitation in stroke care and research. They gauge health domains that are valued by the broader population rather than specifically addressing the concerns of stroke patients (11). Recent progress in research has brought to light notable disparities between patient preferences for health conditions and those of the broader population. As a result, the utilization of generic tools for evaluating QOL in the context of stroke has proven to be insufficient (12). Consequently, there is a pressing need for QOL utility measures tailored specifically to the unique challenges posed by stroke.

The Stroke-Specific Quality of Life Scale (SS-QOL) emerged as a pioneering HRQOL assessment tool exclusively designed for individuals who have experienced a stroke (13). Introduced in 1999, its dual purpose was to quantitatively assess the multifaceted influences of stroke and evaluate the effectiveness of interventional management. Comprising an extensive 49-item assessment, the SS-QOL encompasses a comprehensive range of domains, including mobility, cognition, mood, functionality, and social roles, from which a total QOL score and 12 subscale scores are derived (13). However, despite its initial design, previous researches have yielded varying solutions for the structure of the SS-QOL, proposing one-, two-, four-, eight-, and 12-domain models (14–17). Consequently, the proposed 12-domain structure lacks robust support from these inconsistent findings, allowing for the flexibility of utilizing diverse sub-scale grades or solely relying on the complete score. Moreover, an additional constraint of the SS-QOL lies in its extensive measurement, rendering it unsuitable for regular implementation in both clinical settings and research due to the substantial administrative burden it imposes.

In an endeavor to achieve a comprehensive and well-balanced measurement while mitigating the length of the assessment, the adoption of abbreviated scales was proposed. An instance of such abbreviated scales is the short form of the Stroke Impact Scale (SF-SIS), which consists of eight items intended to evaluate functional and QOL measures (18). Nonetheless, the SF-SIS exhibits limitations in its evaluative scope because it fails to explicitly measure physical and psychosocial functions. The short 17-item form of the SS-QOL (SS-QOL-17) scale was recently introduced to assess a 3-factor model of SS-QOL encompassing the functional and psychosocial domains, supplemented by a novel therapeutic domain (19). By employing the SS-QOL-17, it becomes possible to evaluate not only the functional and psychosocial performance in daily living but also the therapeutic attributes. Although the Arabic version of the SS-QOL-17 demonstrates apparent validity and reliability, it is still advisable to validate the effectiveness of this scale in diverse cultural and linguistic contexts.

Given the potential complications and consequences associated with strokes, the need arises for a considerably condensed utility measure that can offer enhanced convenience in both clinical practice and research. A prime example of such brevity is found in the 12 items version of the SS-QOL scale (SS-QOL-12), as suggested by Post et al. (20). At present, this represents the shortest among the scales associated with SS-QOL. The development of SS-QOL-12 was based on a 2-factor model derived from the original 49-item SS-QOL scale. The item displaying the highest item-total correlation within each of the 12 domains was selected, resulting in the formation of two distinct physical and psychosocial domains. The SS-QOL-12 demonstrated a high correlation with the original SS-QOL, while both its individual subscales and the whole scale exhibited good internal consistency (20). Despite these promising structural and reliability features, a comprehensive investigation into the psychometric properties of the SS-QOL-12 remains warranted, and it is therefore recommended that further scrutiny be devoted to evaluating additional measures of its validity.

On the other hand, the Arabic version of the long 49-item SS-QOL (SSQOL-A) has demonstrated good validity measures and psychometric properties (21). Nevertheless, it cannot be automatically assumed that the Arabic version of SS-QOL-12 would exhibit the same measurement properties as the SSQOL-A. The omission of certain items within the shortened version carries the potential risk of compromising one or more pivotal facets of the original scale, thus engendering dissimilar measurement properties between the abbreviated version and the original scale (22). Consequently, the measurement properties observed in the SSQOL-A may not be readily generalized to the SS-QOL-12, necessitating further in-depth investigations prior to endorsing the utilization of the SS-QOL-12 in Arabic-speaking nations. The aim of the current study was to validate the Arabic version of the SS-QOL-12 (SS-QOL-12-AR) and examine its validity measures and psychometric properties. Additionally, the study aimed to assess the QOL after stroke and identify the sociodemographic and clinical factors that influence it in Lebanon.

## 2. Materials and methods

### 2.1. Study design and participants

This cross-sectional study encompassed individuals who had undergone a stroke event and subsequently survived. Stroke survivors were defined as patients who had previously experienced hospitalization following a stroke and then were discharged alive, irrespective of the magnitude of ensuing problems (23). The process of participants recruitment involved selecting patients from the major community pharmacies dispersed all over districts within Lebanon. All patients and their caregivers who sought medications aimed at secondary prevention of an acute cerebrovascular accident or management of comorbidities known to constitute factors of risk for a stroke (e.g., dyslipidemia, cardiac arrhythmia, hypertension, and diabetes mellitus) were subjected to screening (24). Additionally, individuals who were explicitly recognized with a history of stroke within the medical reports maintained by the pharmacies underwent further screening. The inclusion criteria comprised adult patients aged 18 years and older who had a diagnosis of ischemic or hemorrhagic stroke from a neurologist. Conversely, patients who were suspected to have stroke symptoms without a confirming clinical diagnosis or a preceding hospital admission for stroke were precluded.

Proficient health care practitioners conducted interviews with the included individuals in person or through phone calls. In cases where patients had communication disabilities, the direct caregiver assumed the role of respondent. The dialog commenced with a comprehensive outline, elucidating the investigation’s aims and emphasizing the importance of its results in relation to the QOL experienced by stroke survivors. To facilitate data collection, a meticulously structured questionnaire was devised, employing the Arabic language that serves as the native tongue of Lebanon. The data collection phase spanned from October 2021 to June 2022. On average, approximately 20 min were required to complete the interview and duly record the respondents’ answers.

### 2.2. Scales and variables

The research tool comprised a questionnaire organized into three distinct parts. The first part covered the sociodemographic characteristics including age, gender, residential area, social history, educational background, employment status, and household income. The second part delved into the patients’ current clinical features, medications usage and adherence, stroke history, and past medical history. In order to assess the level of adherence to medications prescribed after a stroke, this section employed the Lebanese Medication Adherence Scale (LMAS-14), a widely accepted generic tool that is validated to evaluate the extent to which stroke patients adhere to their prescribed pharmacotherapy. The LMAS-14 items evaluate adherence levels on a scale encompassing the response options from “never” to “always,” with higher scores indicative of better medication adherence (25). In our sample, the LMAS-14 had a Cronbach’s alpha of 0.928.

The third section of the questionnaire incorporated validated stroke scales that assess stroke prognosis, daily activities and performance, and QOL. To evaluate stroke outcomes and prognosis, the modified Rankin Scale (mRS) was utilized. The mRS employs a single 7-point scale to rate stroke outcomes on hospital discharge, ranging from the absence of symptoms (indicative of the mildest severity) to mortality (indicative of the most severe outcome). Poor prognosis is indicated by dichotomizing the scale score at a cut-off point of 3 or higher (26). Furthermore, the Barthel Index (BI) was integrated into this section as a prognostic tool for post-stroke assessment of daily performance and activities. The BI evaluates 10 functional daily tasks, scoring the level of independence in areas such as feeding, dressing, grooming, bathing, transfers, mobility, stairs, and toileting. Scores within the range of 0–20 indicate total dependency, 21–60 indicate severe dependency, 61–90 indicate moderate dependency, and 91–99 indicate slight dependency (27). The BI had a Cronbach’s alpha of 0.956 within our sample. Lastly, this section included the following SS-QOL scales:

#### 2.2.1. SSQOL-A

The SSQOL-A is a validated Arabic adaptation of the original full-length SS-QOL scale, evaluating identical 49 items and 12 domains of post-stroke aspects linked to energy, upper extremity function, work productivity, mood, self-care, social roles, family roles, vision, language, thinking, and personality. The SSQOL-A generates scores ranging from 49 to 245, where higher scores signify a better QOL (21). In our sample, the Cronbach’s alpha for the SSQOL-A was 0.979.

#### 2.2.2. SS-QOL-17

The SS-QOL-17 is a novel concise scale that was developed and validated in Arabic to cover a comprehensive assessment of post-stroke QOL across three domains that are derived from the BI, SSQOL-A, and LMAS-14. The functional domain, derived from the BI, assesses patients’ daily performance on feeding, bathing, grooming, dressing, toileting, and mobility. The psychosocial domain, derived from the SSQOL-A, assesses aspects of QOL related to energy, family engagement, speech troubles, future discouragement, mood, cognition, social engagement, and general performance. The therapeutic domain, derived from the LMAS-14, assesses medication forgetfulness and unwillingness to receive therapy, primarily due to financial considerations. The final SS-QOL-17 score can range from 17 to 70, with higher scores indicative of a better QOL (19). The Cronbach’s alpha of the SS-QOL-17 among our sample was 0.903.

#### 2.2.3. SS-QOL-12-AR

The SS-QOL-12-AR adopts a two-domain structure and incorporates 12 selected items derived from the original 49-item SSQOL-A scale, following the framework established by Post and colleagues (20). The psychosocial domain includes seven items involving thinking, family roles, social roles, personality, mood, energy, and language. Additionally, the physical domain comprises five items addressing selfcare, mobility, upper extremity function, vision, and work. The initial six items of the SS-QOL12-AR (items 1 to 6) are in a question format, while the remaining six items (items 7 to 12) are positively phrased. Each of the 12 items is assessed using a consistent five-point scale that aligns with its corresponding item in the original SSQOL-A scale. For items 2, 4, 5, 7, 8, 9, 10, 11, and 12, the 5-point response scale encompasses the following options: strongly agree (1), moderately agree (2), neither agree nor disagree (3), moderately disagree (4), and strongly disagree (5). Regarding item 1, respondents’ answers can span the spectrum from total help (1), a lot of help (2), some help (3), a little help (4), to no help needed (5). Finally, responses to items 3 and 6 are measured on a scale that ranges from could not do it at all (1), a lot of trouble (2), some trouble (3), a little trouble (4), to no trouble at all (5). Responses from each item are summed up, and the resulting composite score ranges from a minimum of 12 to a maximum of 60, with higher scores indicating a better QOL.

### 2.3. Ethical aspects

The research protocol received approval from the Ethics and Research Committee of the School of Pharmacy at the Lebanese International University (protocol number: 2020RC-048-LIUSOP). As the study was observational and did not involve any clinical interventions, the requirement for written informed consent was waived. Throughout the data collection and analysis phases, no personal identifiers were recorded or traced, thereby guaranteeing anonymity and maintaining the confidentiality of all participants.

### 2.4. Sample size calculation

The calculation of the minimum sample size utilized the Centers for Disease Control and Prevention Epi-Info software version 7.2.4. The stroke prevalence in Lebanon is reportedly around 3.9% (28). Consequently, a sample size of at least 58 stroke patients was calculated to analyze stroke data. Furthermore, in order to validate the SS-QOL-12-AR scale, it is advisable to maintain a participant-to-item ratio of no less than 10 to 1 (29). Hence, a total of at least 120 patients were essential to validate the scale. Ultimately, the study necessitated a minimal sample size of 120 patients to ensure adequate statistical analysis and achieve a 95% confidence level with a tolerable margin of error = 5%.

### 2.5. Statistical analysis

The data were analyzed using IBM SPSS version 26.0. Sociodemographic and clinical characteristics of the patients were assessed through descriptive statistics. Continuous variables were represented as means and standard deviations (±SD), while categorical variables were expressed as frequencies and percentages.

To validate the structure of the SS-QOL-12-AR, factor analysis was executed utilizing principal component analysis with a rotated matrix. Adequacy of the sample size was confirmed by the Kaiser-Meyer-Olkin (KMO) measure, and the suitability of the data for factor analysis was established by Bartlett’s test of sphericity. Factors with eigenvalues exceeding one were extracted from the final scale, which were then employed to derive the overall SS-QOL-12-AR score. Pearson correlation was used to examine the correlation between each scale item and the overall scale. Internal consistency (reliability) of the scale and factor subscales were assessed via Cronbach’s alpha. The sensitivity and specificity of the SS-QOL-12-AR, SS-QOL-17, and SSQOL-A scales were identified using ROC curve analysis, with the optimal cutoff points identified based on the J-index. Pearson correlation was additionally utilized to assess the convergent validity of the SS-QOL scales.

The SS-QOL-12-AR score was dichotomized into lower and better QOL using the identified optimal cut-off point to determine the proportion of patients with reduced post-stroke QOL. Bivariate analysis, with the dichotomized SS-QOL-12-AR score as the dependent variable, involved conducting chi-square analysis. Subsequently, a multivariable binomial logistic regression was performed to identify predictors of QOL after a stroke, considering prospective confounding factors. Two initial models were constructed with the dichotomized SS-QOL-12-AR score as the dependent variable and variables with *p* values lower than 0.2 from the bivariate analysis as independent variables. The first model included clinical features, while the second incorporated sociodemographic features of the stroke survivors. Afterward, a third model was performed using the clinical and sociodemographic significant variables from the first and second models as independent variables and the dichotomized SS-QOL-12-AR score as the dependent variable. The findings were reported as adjusted odds ratios (ORa) with a 95% confidence interval (CI). A significance level of *p* < 0.05 was employed, with a permissible margin of error of 5%.

## 3. Results

### 3.1. Sociodemographic characteristics

A total of 172 post-stroke patients were included in this study. Greater than half of participants (61.6%) were males, 38.4% were ex-smokers, 70.9% were non-alcoholic, and 45.3% were from the capital Beirut. Furthermore, 46.5% had a school level of education, 43.0% were unemployed, and 27.9% had a monthly household income ranging between 2,000,000 to 3,500,000 Lebanese Pounds (LBP). For marital status, the majority (70.9%) were married, 90.7% were living with a family, and 45.1% had 3 to 4 children. The mean age of patients was 62.67 (±13.38) years, and their mean body mass index (BMI) was 26.81 (±3.40). The detailed sociodemographic characteristics of patients are shown in Table 1.

**Table 1:** Sociodemographic characteristics of the patients.

Variable	Category	Stroke survivors (*N* = 172)	
		Mean (SD)	Frequency (%)
*Gender*	Female		66 (38.4)
	Male		106 (61.6)
*Age*		62.67 (13.38)	
BMI^*^		26.81 (3.40)	
*Smoking status*	Non-smoker		44 (25.6)
	Ex-smoker		66 (38.4)
	Current smoker		62 (36.0)
*Alcohol consumption*	No		122 (70.9)
	In the past, not anymore		36 (20.9)
	Yes, currently		14 (8.1)
*Level of education*	University level		44 (25.6)
	School level		80 (46.5)
	Illiterate		48 (27.9)
*Employment*	Unemployed		74 (43.0)
	Employed		46 (26.7)
	Retired		52 (30.2)
*Monthly household income*	<2,000,000 LBP		40 (23.3)
	2,000,000–3,500,000 LBP		48 (27.9)
	3,500,000–5,000,000 LBP		32 (18.6)
	5,000,000–6,500,000 LBP		22 (12.8)
	6,500,000–8,000,000 LBP		6 (3.5)
	>8,000,000 LBP		24 (14.0)
*Area of residence*	Beirut		78 (45.30)
	Bekaa		12 (7.0)
	Mount Lebanon		40 (23.30)
	North		14 (8.10)
	South		28 (16.30)
*Marital status*	Single		22 (12.8)
	Married		122 (70.9)
	Divorced		8 (4.7)
	Widowed		20 (11.6)
*Number of children (N = 142)*	1 to 2		32 (22.5)
	3 to 4		64 (45.1)
	More than 4		46 (32.4)
*Living with:*	Family (nuclear or extended)		156 (90.7)
	Alone		16 (9.3)

^*^BMI, body mass index.

### 3.2. Clinical characteristics

Table 2 reports the clinical characteristics of the stroke survivors. The majority of strokes (79.1%) were ischemic in nature and the majority of stroke survivors (83.7%) had only encountered a single stroke event. Greater than half of the patients (66.2%) had their stroke within the past 1 to 5 years. On average, the mean number of comorbidities among the patients was 3.80 (±2.44), the mean number of prescribed medications was 6.23 (±3.10), and LMAS-14 mean score was 34.92 (±8.78). In terms of stroke outcomes, approximately three-fourths of the patients (76.7%) exhibited poor prognosis according to the mRS scale, and 46.5% experienced severe to total dependency on the BI scale.

**Table 2:** Clinical characteristics of the stroke survivors.

Variable	Category	Stroke survivors (*N* = 172)	
		Mean (SD)	Frequency (%)
*Type of stroke*	Ischemic		136 (79.1)
	Hemorrhagic		36 (20.9)
*Recurrent stroke*	No		144 (83.7)
	Yes		28 (16.3)
*Date of stroke diagnosis*	Less than 1 year		34 (19.8)
	1 to 5 years		114 (66.2)
	More than 5 years		24 (14.0)
*Number of comorbidities*		3.80 (2.44)	
*Number of prescribed medications*		6.23 (3.10)	
LMAS-14^*^ score		34.92 (8.78)	
mRS^*^	Poor prognosis		132 (76.7)
	Good prognosis		40 (23.3)
BI^*^ classes	Total dependency		36 (20.9)
	Severe dependency		44 (25.6)
	Moderate dependency		28 (16.3)
	Slight dependency		64 (37.2)

^*^LMAS-14, Lebanese Medication Adherence Scale; mRS, modified Rankin Scale; BI, Barthel index.

### 3.3. Validation of the SS-QOL-12-AR

#### 3.3.1. Factor analysis

Factor analysis with PCA was conducted to assess the structure validity of the SS-QOL-12-AR scale for measuring post-stroke QOL. All 12 items could be extracted with Promax rotation. None of the variables exhibited low factor loading (<0.3), low communality (<0.3), or over-correlation (>0.9). The Kaiser-Meyer-Olkin (KMO) measure of sampling adequacy was 0.850, indicating an adequate model. Additionally, the Bartlett’s test of sphericity was highly significant (*p* < 0.001).

The analysis revealed a solution of two factors with Eigenvalues greater than one, and explaining 63.56% of the total variance. Factor 1 comprised 7 items capturing the psychosocial domain factors, with factor loading ranging from 0.545 to 0.805. On the other hand, factor 2 comprised 5 items related to the physical domain factors, with factor loading ranging from 0.575 to 0.812. The Promax rotated matrix of the SS-QOL-12-AR is reported in Table 3.

**Table 3:** Promax rotated matrix of the SS-QOL-12-AR.

Factor	Domain	Factor 1	Factor 2	Communalities
I felt I was a burden to my family.	Family roles	0.805		0.717
I was discouraged about my future.	Mood	0.791		0.626
My physical condition interfered with my social life.	Social roles	0.732		0.665
I was too tired to do what I wanted to do.	Energy	0.683		0.497
I had trouble remembering things.	Thinking	0.630		0.491
My personality has changed.	Personality	0.610		0.622
Did you have to repeat yourself so others could understand you?	Language	0.545		0.558
Did you have trouble seeing the television well enough to enjoy a show?	Vision		0.812	0.684
Did you have trouble buttoning buttons?	Upper extremity function		0.740	0.765
Did you need help taking a bath or shower?	Selfcare		0.660	0.779
Did you have to stop and rest more than you would like when walking or using a wheelchair?	Mobility		0.603	0.576
Did you have trouble doing daily work around the house?	Work		0.575	0.648
*Percentage of variance explained*		53.72%	9.86%	
*Cronbach’s alpha*		0.877	0.830	

Factor 1, Psychosocial (family roles, mood, social roles, energy, thinking, personality, and language); Factor 2, Physical (vision, upper extremity function, selfcare, mobility, work). Cronbach’s alpha for the SS-QOL-12-AR = 0.917. Total percentage of variance explained: 63.56%. Kaiser-Meyer-Olkin (KMO) = 0.850. Bartlett’s test of sphericity: *p* < 0.001.

#### 3.3.2. Psychometric properties

The SS-QOL-12-AR had a high internal consistency for assessing post-stroke QOL indicated by a Cronbach’s alpha of 0.917. The psychosocial and physical subscales also demonstrated high Cronbach’s alpha of 0.877 and 0.830, respectively (Table 3). All items of the SS-QOL-12-AR scale exhibited highly significant correlations with the full scale (*p* < 0.001). The Pearson correlation coefficients ranged from 0.424 to 0.866. The correlations of the SS-QOL-12-AR items with the full scale are shown in Table 4.

**Table 4:** Pearson correlation of the SS-QOL-12 items with the full scale.

SS-QOL-12-AR item number	Items	r^*^	*p*-value
1	Did you need help taking a bath or shower?	0.866	<0.001
2	Did you have to stop and rest more than you would like when walking or using a wheelchair?	0.674	<0.001
3	Did you have trouble buttoning buttons?	0.822	<0.001
4	Did you have to repeat yourself so others could understand you?	0.750	<0.001
5	Did you have trouble seeing the television well enough to enjoy a show?	0.424	<0.001
6	Did you have trouble doing daily work around the house?	0.795	<0.001
7	I had trouble remembering things.	0.742	<0.001
8	I felt I was a burden to my family.	0.790	<0.001
9	My physical condition interfered with my social life.	0.785	<0.001
10	My personality has changed.	0.785	<0.001
11	I was discouraged about my future.	0.599	<0.001
12	I was too tired to do what I wanted to do.	0.638	<0.001

^*^Pearson correlation coefficient.

**Figure 1:**
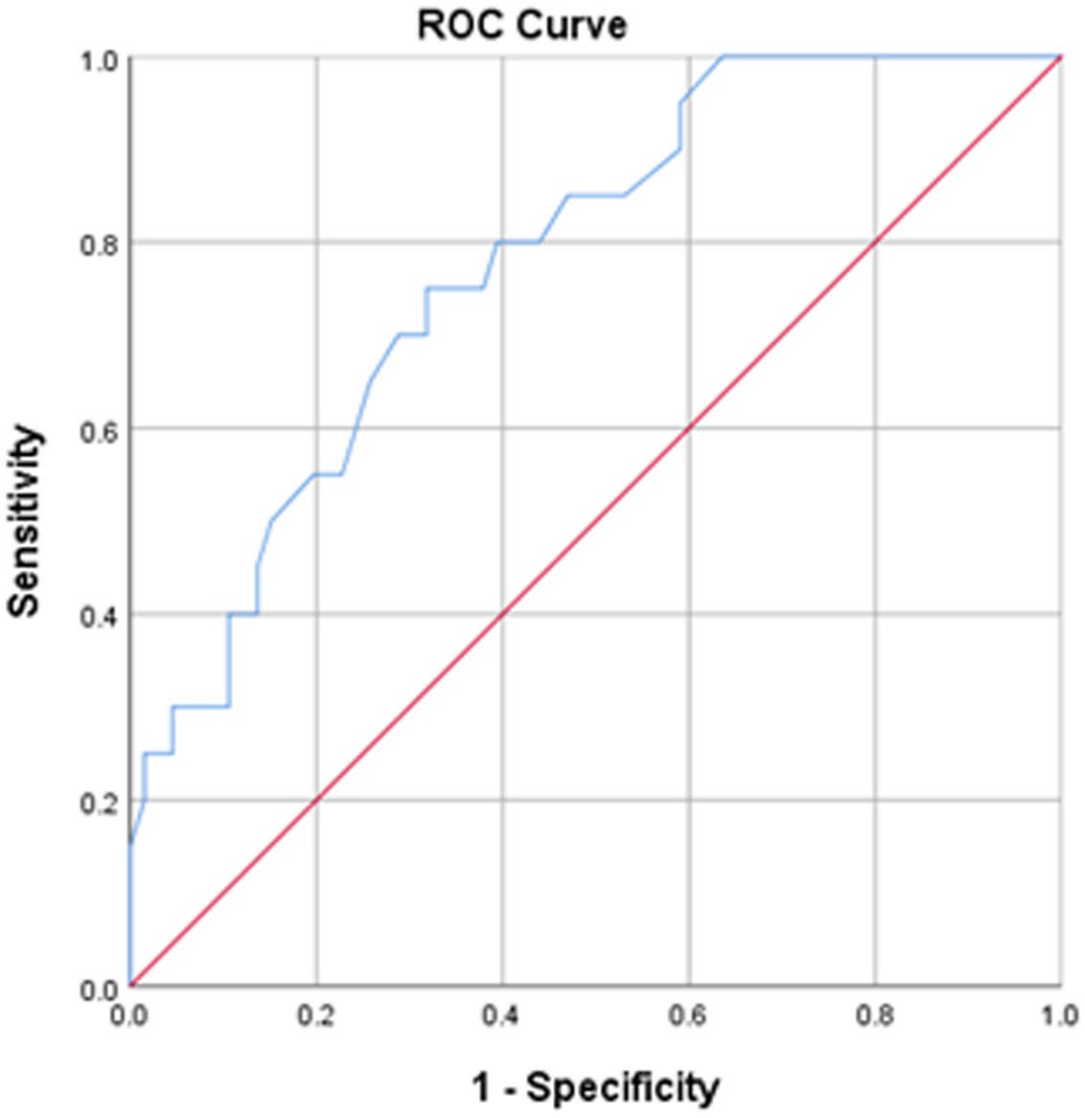
ROC curve of the SS-QOL-12-AR. Stroke survivors with good prognosis on mRS at hospital discharge were analyzed. Area under the curve = 0.779; 95% CI 0.704–0.855 (*p* < 0.001). At value = 32.50, sensitivity = 70.00% and specificity = 71.20%.

The SS-QOL-12-AR had a mean of 30.10 (±12.87) with higher values indicating better QOL. Figure 1 presents the ROC curve analysis of the SS-QOL-12-AR comparing post-stroke patients with good prognosis on the mRS to those with poor prognosis. An optimal cut-off point of 32.50 was identified for determining a better QOL. This cut-off point demonstrated a sensitivity of 70.00% and a specificity of 71.20%. The area under the curve (AUC) was calculated as 0.779; 95% CI 0.704–0.855 (*p* < 0.001).

**Figure 2:**
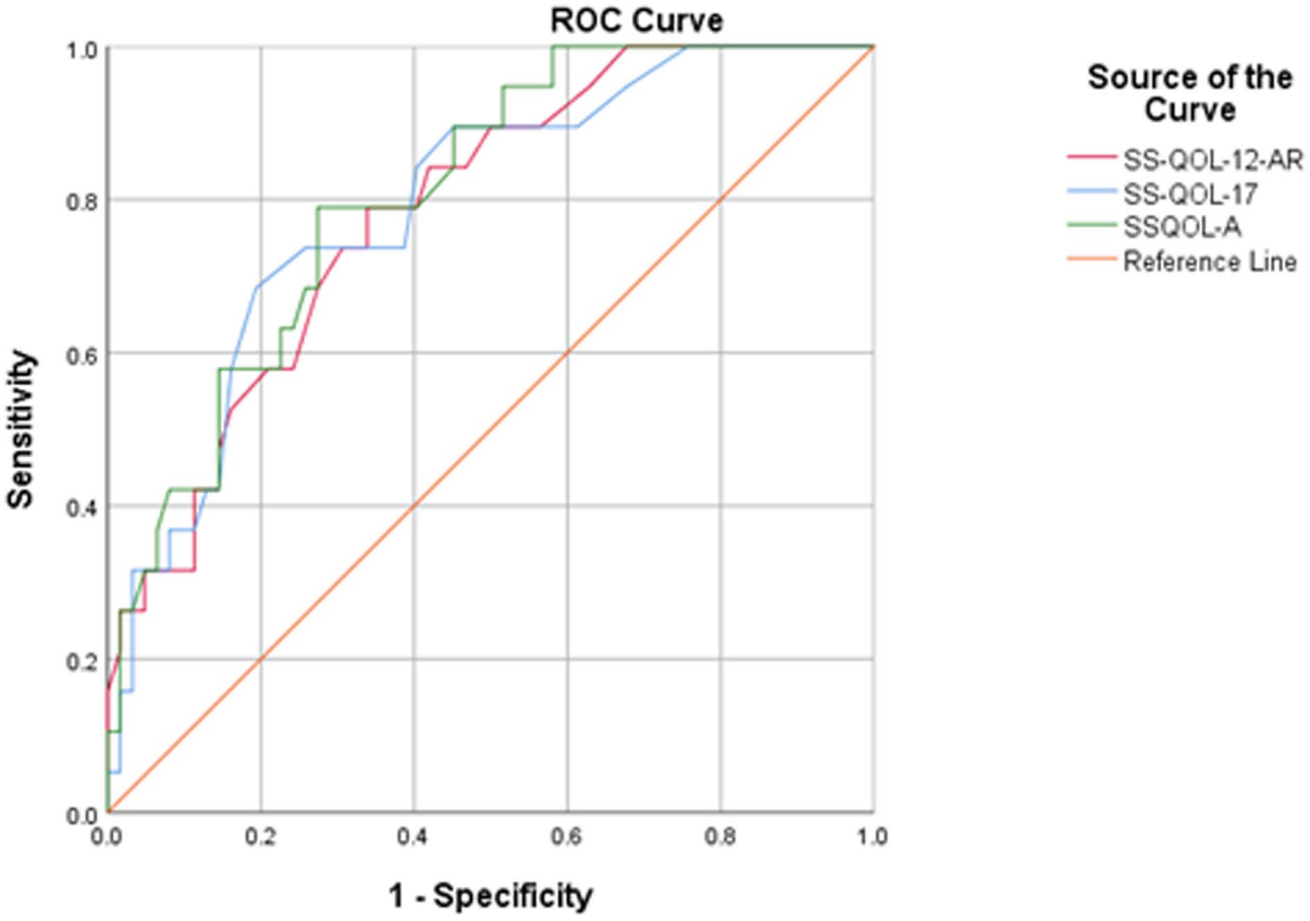
Comparison of ROC curves of the SS-QOL-12-AR, SS-QOL-17, and SSQOL-A. Stroke survivors with good prognosis on mRS at hospital discharge were analyzed. SS-QOL-12-AR: area under the curve = 0.779; 95% CI 0.704–0.855 (*p* < 0.001). At value = 32.50, sensitivity = 70.00% and specificity = 71.20%. SS-QOL-17: area under the curve = 0.787; 95% CI 0.712–0.863 (*p* < 0.001). At value = 44.00, sensitivity = 70.00% and specificity = 75.80%. SSQOL-A: area under the curve = 0.807; 95% CI 0.736–0.879 (*p* < 0.001). At value = 133.50, sensitivity = 78.90% and specificity = 72.60%.

Figure 2 presents the ROC curve analyses comparing SS-QOL-12-AR, SS-QOL-17, and SSQOL-A. Stroke survivors with good prognosis on the mRS were also analyzed. The SS-QOL-17 had an area under the curve of 0.787; 95% CI 0.712–0.863 (*p* < 0.001). The sensitivity and specificity of the SS-QOL-17 for determining a better QOL were 70.00 and 75.80% respectively, at a cut-off value of 44.00. The area under the curve of the SSQOL-A was 0.807; 95% CI 0.736–0.879 (*p* < 0.001). At a cut-off value of 133.50, the SSQOL-A had a sensitivity of 78.90% and specificity of 72.60% for determining better QOL among stroke survivors.

#### 3.3.3. Convergent validity

The SS-QOL-12-AR total score was significantly correlated with the total scores of the other versions of the SS-QOL scales (SS-QOL-17: Pearson correlation coefficient = 0.939, *p* < 0.001; and SSQOL-A: Pearson correlation coefficient = 0.983, *p* < 0.001). The total scores of the SS-QOL-17 and SSQOL-A were also significantly correlated (Pearson correlation coefficient = 0.956, *p* < 0.001).

### 3.4. Predictors of post-stroke QOL

#### 3.4.1. Bivariate analysis

The SS-QOL-12-AR score was dichotomized at the cut-off value revealed by the ROC curve analysis (32.50) to compare stroke survivors and determine factors that are associated with a lower or better QOL. 61.6% of stroke survivors had a lower QOL. Analysis of the sociodemographic characteristics showed that older age was significantly associated with a lower QOL (*p* = 0.023). There was also a significant difference between QOL according to the smoking status (*p* < 0.001), alcohol consumption (*p* < 0.001), level of education (*p* = 0.002), and area of residence (*p* = 0.001).

Analysis of the clinical characteristics showed a significantly lower QOL with a higher mean number of comorbidities (*p* = 0.004) and a lower mean score of LMAS-14 (*p* = 0.006). There was also a significant difference between QOL according to the mRS prognosis status (<0.001) and BI classes (<0.001). The bivariate analysis comparing stroke survivors with lower and better QOL with their sociodemographic and clinical characteristics are reported in Table 5.

**Table 5:** Bivariate analysis comparing stroke survivors with lower and better QOL with their sociodemographic and clinical characteristics.

Variable	Lower QOL *N* = 106 (61.6%)		Better QOL *N* = 66 (38.4%)		*p*-value
Category	Frequency (%)	Mean (SD)	Frequency (%)	Mean (SD)	
**Gender**					0.389
Female	38 (37.60)		28 (42.40)		
Male	68 (64.2)		38 (35.8)		
**Age**		64.62 (11.66)		59.55 (15.33)	0.023
BMI^*^		26.73 (3.17)		26.95 (3.78)	0.697
**Smoking status**					<0.001
Non-smoker	16 (36.4)		28 (63.6)		
Ex-smoker	50 (75.8)		16 (24.2)		
Current smoker	40 (64.5)		22 (35.5)		
**Alcohol consumption**					<0.001
No	72 (59.0)		50 (41.0)		
In the past, not anymore	30 (83.3)		6 (16.7)		
Yes, currently	4 (28.6)		10 (71.4)		
**Level of education**					0.002
University level	18 (40.9)		26 (59.1)		
School level	58 (72.5)		22 (27.5)		
Illiterate	30 (62.5)		18 (37.5)		
**Area of residence**					0.001
Beirut	42 (53.8)		36 (46.2)		
Bekaa	8 (66.7)		4 (33.3)		
Mount Lebanon	28 (70)		12 (30)		
North	14 (100)		0 (0)		
South	14 (50)		14 (50)		
**Marital status**					0.623
Single	12 (54.5)		10 (45.5)		
Married	74 (60.7)		48 (39.3)		
Divorced	6 (75)		2 (25)		
Widowed	14 (70)		6 (30)		
**Number of children (*N* = 142)**					0.492
1 to 2	22 (68.8)		10 (31.3)		
3 to 4	36 (56.3)		28 (43.8)		
More than 4	28 (60.9)		18 (39.1)		
**Living with**					0.583
Family (nuclear or extended)	96 (61.5)		60 (38.5)		
Alone	10 (62.5)		6 (37.5)		
**Type of stroke**					0.1
Ischemic	80 (58.8)		56 (41.2)		
Hemorrhagic	26 (72.2)		10 (27.8)		
**Recurrent stroke**					0.463
No	88 (61.1)		56 (38.9)		
Yes	18 (64.3)		10 (35.7)		
**Date of stroke diagnosis**					0.068
Less than 1 year	20 (58.8)		14 (41.2)		
1 to 5 years	76 (66.7)		38 (33.3)		
More than 5 years	10 (41.7)		14 (58.3)		
**Number of comorbidities**		4.23 (2.46)		3.12 (2.26)	0.004
LMAS-14^*^score		33.57 (9.42)		37.09 (7.18)	0.006
mRS^*^					<0.001
Poor prognosis	94 (71.2)		38 (28.8)		
Good prognosis	12 (30.0)		28 (70.0)		
BI^*^classes					<0.001
Total dependency	34 (94.4)		2 (5.6)		
Severe dependency	42 (95.5)		2 (4.5)		
Moderate dependency	14 (50.0)		14 (50.0)		
Slight dependency	16 (25.0)		48 (75.0)		

^*^BMI, Body Mass Index; LMAS-14, Lebanese Medication Adherence Scale; mRS, modified Rankin Scale; BI, Barthel Index.

#### 3.4.2. Multivariable analysis

Three models of multivariable binomial logistic regression were performed taking the SS-QOL-12-AR dichotomized score as the dependent variable. The first model included the clinical characteristics of the stroke survivors as independent variables. Poor stroke prognosis on the mRS was significantly associated with lower QOL (ORa = 0.274, *p* = 0.021). Lower QOL was also significantly associated with moderate physical dependence (ORa = 0.312, *p* = 0.042), severe physical dependence (ORa = 0.013, *p* < 0.001), and total physical dependence (ORa = 0.010, *p* < 0.001) compared to slight physical dependence on the BI.

The second model included the sociodemographic characteristics of the stroke survivors as independent variables. Stroke survivors who never smoked had significantly better QOL compared to current smokers (ORa = 4.565, *p* = 0.002). While non-alcoholic patients (ORa = 0.189, *p* = 0.019) and previously alcoholic patients (ORa = 0.088, *p* = 0.003) had a significantly lower QOL compared to stroke survivors with current alcohol consumption.

The third model included the clinical and sociodemographic characteristics of stroke survivors that were statistically significant in the first and second models as independent variables. Poor stroke prognosis on the mRS remained significantly associated with lower QOL (ORa = 0.361, *p* = 0.037). Lower QOL was also significantly associated with severe physical dependence (ORa = 0.092, *p* < 0.001) and total physical dependence (ORa = 0.018, *p* < 0.001) compared to slight physical dependence on the BI. On the other hand, stroke survivors who never smoked had significantly better QOL compared to current smokers (ORa = 3.782, *p* = 0.004). The multivariable logistic regression taking the SS-QOL-12-AR score as the dependent variable is presented in Table 6.

**Table 6:** Multivariable logistic regression taking the SS-QOL-12-AR dichotomized score as the dependent variable.

Variable	Adjusted odds ratio	95% confidence interval		*p*-value
		Lower	Upper	
Model 1^*^including clinical characteristics				
*mRS^‡^ (poor vs. good prognosis)*	0.274	0.091	0.822	0.021
***BI***^***‡***^ ***classes (Reference: slight dependency)***				
Moderate dependency	0.312	0.102	0.957	0.042
Severe dependency	0.013	0.002	0.073	<0.001
Total dependency	0.01	0.002	0.059	<0.001
Model 2^**^including social characteristics				
*Smoking (non-smoker vs. current smoker)*	4.565	1.749	11.916	0.002
***Alcohol consumption (Reference: yes, currently)***				
In the past, not anymore	0.088	0.018	0.426	0.003
No	0.189	0.047	0.764	0.019
Model 3^***^including statistically significant clinical and social characteristics from models 1 and 2				
*mRS^‡^ (poor vs. good prognosis)*	0.361	0.139	0.938	0.037
***BI***^***‡***^ ***classes (Reference: slight dependency)***				
Moderate dependency	0.557	0.219	1.414	0.218
Severe dependency	0.092	0.033	0.252	<0.001
Total dependency	0.018	0.003	0.093	<0.001
*Smoking (non-smoker vs. current smoker)*	3.782	1.695	8.443	0.004

^*^Variables initially included in the model: type of stroke; date of stroke diagnosis; number of comorbidities; Lebanese Medication Adherence Scale (LMAS-14).

^**^Variables initially included in the model: age; level of education; area of residence.

^***^Variables initially included in the model: alcohol consumption.

^‡^mRS, modified Rankin Scale; BI, Barthel Index.

## 4. Discussion

This study validated the Arabic version of the short 12-item stroke-specific QOL scale (SS-QOL-12-AR), with the aim to comprehensively examine the psychometric properties of this scale and provide additional explicit evidence supporting its applicability to assess QOL among stroke survivors. Our findings provide obvious evidence to support the scale’s validity and reliability for this purpose. The scale exhibited very good psychometric properties and internal consistency, thus recommending its utilization in both stroke research and clinical practice. The QOL experienced post-stroke was significantly predicted by clinical characteristics associated with stroke prognosis and physical dependence. Moreover, the post-stroke QOL was significantly predicted by sociodemographic factors encompassing the social history of tobacco use and alcohol consumption.

### 4.1. Validation of the SS-QOL-12-AR

The present study successfully validated the SS-QOL-12-AR, a concise scale designed to assess HRQOL following a stroke. This Arabic version of the scale was developed based on the Dutch version introduced by Post and colleagues (20), which in turn drew inspiration from the extensive 49-item SS-QOL/SSQOL-A (13, 21). The ultimate goal was to offer stroke survivors a more streamlined utility measure that would be easier to administer compared to the lengthy and burdensome existing scales. In our sample, the preexisting Arabic version of the longer scale demonstrated satisfactory reliability and internal consistency. However, the practicality of its implementation could be limited by its excessive length and the associated burden on assessment procedures. Therefore, it was imperative to adapt and validate a shorter and simpler scale that would require less time to administer to stroke patients, while still effectively capturing the post-stroke psychosocial and physical dimensions. The development of the SS-QOL-12-AR involved a meticulous process of item selection, wherein one item was carefully selected from each of the SSQOL-A’s 12 domains. The selection criteria were based on the highest correlation of each item with its respective domain, as proposed by Post et al. (20). This methodological framework ensured that the final SS-QOL-12-AR combined the aspects of HRQOL linked to family roles, mood, social roles, energy, thinking, personality, language, vision, upper extremity function, selfcare, mobility, and work.

The outcomes of our psychometric properties analysis revealed that the SS-QOL-12-AR is reliable, as evident from the computed Cronbach’s alpha for the entire scale and factor subscales (30). Employing factor analysis, the SS-QOL-12-AR items were divided into two distinct factors. Factor 1, representing the psychosocial domain, encompassed family roles, mood, social roles, energy, thinking, personality, and language. Factor 2, representing the physical domain, included vision, upper extremity function, selfcare, mobility, and work. Comparing the structure of the SS-QOL-12-AR with the Dutch version proposed by Post and colleagues, we observed a high level of compatibility, with one exception. The factor of “Language,” which was included in the physical domain of the Dutch version, deviated from our findings, where it was associated with the psychosocial factor. The current SS-QOL-12-AR version presents an improved structure of the scale since Post and colleagues adopted a theoretical division of domains based on the long-scale version, without specifically assessing the structure validity of the abbreviated 12-item scale. Moreover, the current result is supported by the findings of Chou and colleagues that determined a very low factor loading (0.150) of “language” on the physical domain of the SS-QOL-12 Taiwanese version (31).

The internal consistency of the SS-QOL-12-AR was excellent when compared to previous Arabic versions of stroke-specific QOL scales. The Cronbach’s alpha for the SS-QOL-12-AR was 0.917, which is comparable to the 49-item SSQOL-A (0.979) and the 17-item SS-QOL-17 (0.903) within the same sample. The reliability evaluations conducted in prior validation studies for the 49-item SS-QOL versions have reported Cronbach’s alpha values ranging from 0.810 to 0.970 (32–35). On the other hand, earlier validated versions of the SS-QOL-12 had Cronbach’s alpha values of 0.850 and 0.880 for the Dutch and American versions, respectively (20, 36). Notably, the reproducibility of the SS-QOL-12-AR was confirmed by the highly significant correlations observed between each item of the scale and the overall scale. Although two items (vision and mood) exhibited lower correlations with the overall scale compared to other items (Pearson correlation coefficients of 0.424 and 0.599, respectively), these correlations still fall within the moderate correlation range, as both items maintain a Pearson correlation coefficient exceeding 0.40 (37). Furthermore, the outcomes of the factor analysis reveal that “mood” (item 11) demonstrates a good factor loading of 0.791 within the psychosocial domain (factor 1) of the scale, and “vision” (item 5) also exhibits a strong factor loading of 0.812 within the physical domain (factor 2) of the scale. These findings collectively endorse the inclusion of these two items in the final scale and underscore the construct validity of the SS-QOL-12-AR (38).

The construct validity of the SS-QOL-12-AR was further established through the computation of sensitivity and specificity of the scale, indicating its efficacy in assessing stroke-related QOL (39). However, the comparison of these sensitivity and specificity values with existing literature wasn’t possible due to the absence of previously determined sensitivity and specificity for earlier versions of the SS-QOL-12. Thus, further research is recommended to validate these current findings across diverse populations and languages. While within the same sample, the SS-QOL-12-AR exhibited sensitivity and specificity levels comparable to those of the SSQOL-A and SS-QOL-17. The analysis of ROC curves for the three scales demonstrated comparable sensitivities and specificities of at least 70% and comparable areas under the curve. Furthermore, the measures of convergent validity between the three scales displayed highly significant and strong correlations, all of which provide support for the construct validity of the SS-QOL-12-AR.

### 4.2. Assessment of post-stroke QOL

The current study revealed that a considerable portion of stroke survivors (61.6%) exhibit diminished QOL. These findings align with previous research indicating that less than 50% of patients achieve independence in their daily lives after a stroke (40). Furthermore, even among patients who regain functional independence, significant deficits, limitations, and alterations in cognitive functions and behavior persist (41). The current findings also indicate that a lower QOL is significantly associated with poor stroke prognosis at hospital discharge, and with greater physical dependence. Rangaraju and colleagues previously suggested that stroke prognosis assessed by the mRS and physical dependence measured by the BI may exhibit relative homogeneity, wherein higher mRS scores and greater physical dependence on the BI correlate with lower QOL up to 3 months following a stroke (42). The results of the present study reinforce this hypothesis and make a valuable contribution to the existing literature by highlighting the enduring predictive significance of stroke outcomes on post-stroke QOL. This is particularly remarkable because our sample included a substantial portion of patients who had encountered their stroke event more than 1 year ago.

### 4.3. Predictors of post-stroke QOL

Stroke survivors who have never smoked exhibited significantly higher QOL compared to those who are current smokers. Previous research reported that continued smoking following a stroke has been linked to adverse health outcomes that could consequently correlate with a diminished QOL (43). Nonetheless, it is plausible to consider the smoking-QOL relationship in the opposite direction as well. The persistence of smoking after a stroke may result from a poor QOL, as previous studies have indicated that psychiatric symptoms are associated with lower rates of smoking cessation and an increased risk of smoking relapse (44). Further research is recommended in this context to gain deeper insights into this relationship and identify areas to support smoking cessation programs specifically tailored for stroke survivors. Such programs are crucial for mitigating further stroke consequences and reducing the risk of stroke recurrence. Furthermore, stroke survivors who never consumed alcohol and those who abstained from alcohol consumption exhibited a diminished QOL compared to current alcohol users. This association remains not fully understood, especially since alcohol use is generally associated with a higher risk of stroke and more severe outcomes (45). Several hypotheses can be proposed to explain this finding. Lower QOL among previously alcoholic stroke survivors could be attributed to experiencing a more severe and damaging stroke, leading to the disruption of social habits including alcohol cessation in tandem with poor QOL (46). Whereas, lower QOL among stroke survivors who never consumed alcohol could potentially be linked to their lower socioeconomic status, which may limit their ability to afford regular alcohol consumption as part of their social habits. Notably, prior studies have established a significant correlation between stroke survivors with a lower socioeconomic status and a diminished QOL following a stroke (47, 48). Additional research is also suggested in this context to gain better understanding of this association.

### 4.4. Study implications

The existing 49-item SS-QOL measurement tools, including the SSQOL-A, are often lengthy and burdensome to administer, and their structural validity results have been inconsistent. Although the original SS-QOL scale has been validated and currently serves as the established standard for assessing post-stroke QOL, validation attempts across different languages and cultures have yielded variable results. Consequently, one of the primary motivations for developing and validating shorter scales was to address this issue. It is worth noting that almost all of the abbreviated SS-QOL scale versions were developed on the basis of the original 49-item SS-QOL. Therefore, the SS-QOL/SSQOL-A scale continues to be widely accepted globally as the reference scale for comparing the validity outcomes of all newly developed abbreviated scales. The findings of the present study validate a considerably shorter version, the SS-QOL-12-AR, which can effectively support stroke research and care systems. The SS-QOL-12-AR demonstrates its effectiveness as a very good measuring tool for assessing stroke-related QOL, making it highly recommended for both stroke research and clinical practice. The concise structure of this scale allows for the evaluation of psychosocial and physical aspects of daily living after surviving a stroke, while radically reducing the burden and complexity of assessment. Nevertheless, further research is advised to confirm the scale’s validity across additional cultures and languages. Moreover, our study’s notable findings regarding the correlation between clinical factors (such as stroke outcomes) and sociodemographic factors (such as social habits) with stroke-related QOL underscore the importance of further comprehensive rehabilitation plans. These plans should integrate psychosocial and physical improvements alongside clinical and social indicators in order to enhance the QOL for stroke survivors.

### 4.5. Strengths and limitations

While stroke prevalence is relatively low in Lebanon, the current study effectively included the necessary sample size, thereby ensuring sufficient power for all statistical analyses. Moreover, the sample encompassed patients from various districts across Lebanon, thereby minimizing the risk of selection bias associated with different QOL indicators among individuals from diverse societal backgrounds. However, it is important to acknowledge several limitations. Firstly, there is not an authenticated gold standard for choosing a criterion validity measure for QOL after a stroke. In this study, the mRS was chosen to assess the validity of the SS-QOL-12-AR, which may be more closely associated with the physical domain of QOL rather than the psychosocial domain. Future research could consider employing generic QOL criterion measures to provide additional evidence of validity, focusing more on the psychosocial aspects. Secondly, this study employed two distinct data collection methods: face-to-face interviews and phone calls. Although this could introduce a potential information bias, it is presumed that the risk is minimal due to the predominant reliance on self-reported responses rather than direct observation of patient reactions. Thirdly, the study design was cross-sectional, which limits the ability to establish causality or determine temporal relationships between sociodemographic and clinical factors, and QOL. Forthcoming studies are suggested to provide longitudinal follow up of stroke outcomes and offer more evidence regarding predictors of QOL. Nonetheless, it should be noted that those studies may continue to carry a possible risk of bias due to the fluctuating nature of stroke outcomes over time, resulting in both positive and negative changes. Lastly, the current multivariable analyses did not include any independent variables on rehabilitation and mental health, so the possibility of residual confounding cannot be precluded. Future research will explore how rehabilitation and different mental health conditions influence the QOL of patients who have experienced a stroke.

## 5. Conclusion

The SS-QOL-12-AR demonstrates excellent psychometric properties, establishing its validity and reliability as a robust tool for assessing the QOL of stroke survivors. Its practicality and benefits make it a valuable instrument for measuring the consequences of stroke in both clinical practice and research settings, enabling the evaluation of management and rehabilitation outcomes. The SS-QOL-12-AR proves to be a useful tool in determining the HRQOL following a stroke, aiding in the identification of patients who require increased attention in their care plan and rehabilitation strategies, potentially uncovering areas for enhancing their QOL. Additional studies are suggested to provide more evidence regarding the validity of the 12-item SS-QOL in other languages and populations. Lastly, this study highlights the significant role of clinical and social factors in predicting post-stroke QOL, emphasizing the importance of recognizing and addressing lower QOL to ultimately optimize outcomes and enhance stroke care and support.

## Data availability statement

The raw data supporting the conclusions of this article will be made available by the authors, without undue reservation.

## Ethics statement

The studies involving humans were approved by the Ethics and Research Committee of the School of Pharmacy at the Lebanese International University. The studies were conducted in accordance with the local legislation and institutional requirements. The ethics committee/institutional review board waived the requirement of written informed consent for participation from the participants or the participants’ legal guardians/next of kin because this study was observational and did not include any interventional procedures on participants.

## Author contributions

FS conceptualized the study, performed investigation, formal analysis and validation, data curation, wrote the original draft, and reviewed and edited the final draft of the manuscript. MD and MA reviewed and edited the manuscript. HH and PS supervised the whole course of the project. All authors contributed to the article and approved the submitted version.
